# Association of Total Knee Replacement Removal From the Inpatient-Only List With Outpatient Surgery Utilization and Outcomes in Medicare Patients

**DOI:** 10.1001/jamanetworkopen.2023.16769

**Published:** 2023-06-05

**Authors:** Derek T. Schloemann, Thomas Sajda, Benjamin F. Ricciardi, Caroline P. Thirukumaran

**Affiliations:** 1 Department of Orthopaedics and Physical Performance, University of Rochester Medical Center, Rochester, New York

## Abstract

**Importance:**

Little is known about the association of total knee replacement (TKR) removal from the Medicare inpatient-only (IPO) list in 2018 with outcomes in Medicare patients.

**Objective:**

To evaluate (1) patient factors associated with outpatient TKR use and (2) whether the IPO policy was associated with changes in postoperative outcomes for patients undergoing TKR.

**Design, Setting, and Participants:**

This cohort study included data from administrative claims from the New York Statewide Planning and Research Cooperative System. Included patients were Medicare fee-for-service beneficiaries undergoing TKRs or total hip replacements (THRs) in New York State from 2016 to 2019. Multivariable generalized linear mixed models were used to identify patient factors associated with outpatient TKR use, and with a difference-in-differences strategy to examine association of the IPO policy with post-TKR outcomes relative to post-THR outcomes in Medicare patients. Data analysis was performed from 2021 to 2022.

**Exposures:**

IPO policy implementation in 2018.

**Main Outcomes and Measures:**

Use of outpatient or inpatient TKR; secondary outcomes included 30-day and 90-day readmissions, 30-day and 90-day postoperative emergency department visits, non–home discharge, and total cost of the surgical encounter.

**Results:**

A total of 37 588 TKR procedures were performed on 18 819 patients from 2016 to 2019, with 1684 outpatient TKR procedures from 2018 to 2019 (mean [SD] age, 73.8 [5.9] years; 12 240 female [65.0%]; 823 Hispanic [4.4%], 982 non-Hispanic Black [5.2%], 15 714 non-Hispanic White [83.5%]). Older (eg, age 75 years vs 65 years: adjusted difference, −1.65%; 95% CI, −2.31% to −0.99%), Black (−1.44%; 95% CI, −2.81% to −0.07%), and female patients (−0.91%; 95% CI, −1.52% to −0.29%), as well as patients treated in safety-net hospitals (disproportionate share hospital payments quartile 4: −18.09%; 95% CI, −31.81% to −4.36%), were less likely to undergo outpatient TKR. After IPO policy implementation in the TKR cohort, there were lower adjusted 30-day readmissions (adjusted difference [AD], −2.11%; 95% CI, −2.73% to −1.48%; *P* < .001), 90-day readmissions ( −3.23%; 95% CI, −4.04% to −2.42%; *P* < .001), 30-day ED visits ( −2.45%; 95% CI, −3.17% to −1.72%; *P* < .001), 90-day ED visits (−4.01%; 95% CI, −4.91% to −3.11%; *P* < .001) and higher cost per encounter ($2988; 95% CI, $415 to $5561; *P* = .03). However, these changes did not differ from changes in the THR cohort except for increased TKR cost of $770 per encounter ($770; 95% CI, $83 to $1457; *P* = .03) relative to THR.

**Conclusions and Relevance:**

In this cohort study of patients undergoing TKR and THR, we found that older, Black, and female patients and patients treated in safety-net hospitals may have had lesser access to outpatient TKRs highlighting concerns of disparities. IPO policy was not associated with changes in overall health care use or outcomes after TKR, except for an increase of $770 per TKR encounter.

## Key Points

**Question:**

Was total knee replacement (TKR) removal from the Medicare inpatient-only (IPO) list in 2018 associated with changes in outcomes in Medicare patients?

**Findings:**

In this cohort study of 37 588 Medicare fee-for-service procedures in a US state administrative database, older, Black, and female patients, as well as patients treated at safety-net hospitals, were less likely to undergo outpatient TKR. When compared with total hip replacements, the IPO policy was not associated with changes in outcomes after TKRs, except for an increase of $770 per encounter.

**Meaning:**

These results suggest that there may be disparities in access to outpatient TKRs; TKR removal from the IPO list resulted in a modest increase in cost for surgical encounters but no difference in postoperative health care utilization.

## Teaser

This cohort study examines rates of surgery and outcomes associated with the removal of total knee replacement from the Medicare inpatient-only list.

## Introduction

Total knee replacement (TKR) is a common procedure for end-stage degenerative disease of the knee, with annual volume in the US estimated at over 680 000 per year in 2014, with a projected increase to 935 000 by 2030.^1,2^ Patients consistently report satisfaction, pain relief, and improved function after TKR.^3,4^

The Centers for Medicare & Medicaid Services (CMS) has historically considered TKR to be an inpatient procedure; however, improvements in perioperative protocols have resulted in shorter hospital stays.^5,6^ Prior studies have demonstrated that outpatient TKR with stays less than 48 hours is safe in carefully selected patients, with no difference in postoperative readmissions, emergency department (ED) visits, or failure to rescue^7,8,9,10,11,12,13,14,15,16^ and with lower risk for medical complications^14,17^ compared with inpatient TKR. These findings contributed to the removal of TKR from the CMS inpatient-only (IPO) list in January 2018, allowing TKR to be performed in the inpatient or outpatient hospital setting^18,19^ and the addition of TKR to the Ambulatory Surgery Center (ASC) Covered Procedures List in January 2020.^20^ The proportion of outpatient TKRs for Medicare beneficiaries increased from 0.2% in 2017 to 36.4% in early 2019,^21^ with savings of $355 million over the first 18 months following policy implementation.^22^ Prior work has shown that this policy has also led to increases in outpatient TKR among privately insured patients.^23^

There are few reports on the use and outcomes of TKR among Medicare patients after IPO policy implementation, especially when compared with a control group to whom the policy did not apply. The study objectives were to evaluate (1) the demographics of patients undergoing outpatient TKR after IPO policy implementation and (2) whether IPO policy was associated with an overall change in postoperative outcomes (eg, 30-day or 90-day readmissions and ED visits, discharge destination, and encounter cost) for patients undergoing TKRs (treatment group) compared with those undergoing total hip replacements (THRs) (control group).

## Methods

The University of Rochester institutional review board approved the study, with a waiver granted for informed consent due to the deidentified nature of the administrative data used. Data analysis was performed from October 2021 to May 2022. This study followed the Strengthening the Reporting of Observational Studies in Epidemiology (STROBE) reporting guideline.

### Data Sources and Study Cohort

We used the inpatient, outpatient, ambulatory surgery, and emergency department files from the New York Statewide Planning and Research Cooperative System (SPARCS) database between 2016 and 2019. SPARCS is a comprehensive all-payer data reporting system that collects patient-level data on patient characteristics, diagnoses and treatment, services, and charges for care in New York State (NYS) health care facilities. The surgical codes used to identify patients undergoing TKR (intervention group) or THR (control group) are in eTable 1 in Supplement 1. Because IPO policy is applicable to Medicare fee-for-service (Medicare) patients only, we limited the study cohort to these patients using primary source of payment typology variable in SPARCS.

We linked the SPARCS facility identifier with American Hospital Association (AHA) facility identifier using the Healthcare Cost and Utilization Project AHA linkage files^24^ to obtain hospital characteristics from the 2016 AHA Annual Survey Database.^25^ We used the 2015 NYS Annual Medicaid Disproportionate Share Hospital (DSH) Report file to obtain DSH data,^26^ the CMS impact file for cost-to-charge ratios needed for estimating costs from total hospital charges,^27^ and Federal Reserve data to convert costs to 2019 US dollars.^28^

The study included Medicare TKR or THR encounters from January 1, 2016, to December 31, 2019. We excluded cases not financed by Medicare fee-for-service, age under 65 years, nonelective admission for fractures or degenerative changes (presumably with severe acute or subacute worsening of symptoms), with non-NYS residency, and unknown gender.

### Outcome Variables

To determine characteristics of patients who were more likely to undergo outpatient TKRs following IPO policy implementation (objective 1), the outcome was a binary indicator of outpatient (outpatient or ambulatory service claim type in SPARCS) or inpatient (inpatient claim type in SPARCS) TKR.

To assess the association of IPO policy implementation with post-TKR outcomes (objective 2), the outcomes were binary indicators for 1 or more readmissions within 30 or 90 days of discharge after the index encounter; 1 or more ED visits within 30 or 90 days of discharge after the index encounter, defined as ED encounter (SPARCS ED claim type) or admission from ED within the time frame of interest after hospital discharge; discharge to a facility vs home; and a continuous indicator for total cost (adjusted to 2019 dollars) for the index encounter estimated from facility charges (*chrg_tot_amt* variable in SPARCS) using the cost-to-charge ratio. We used 2020 SPARCS data to evaluate 30-day and 90-day outcomes for those operations in 2019.

### Key Independent Variables

To examine association of IPO policy with a change in TKR outcomes, the key independent variables were a binary indicator for procedure (THR vs TKR), a binary indicator for policy phase (whether a discharge occurred before or after the January 1, 2018, policy implementation), and an interaction between these variables. We chose patients undergoing THRs as a control group because the recovery and rehabilitation protocols are similar and standardized,^5,6^ and many lower-extremity total joint replacement policies include both procedures.^29^ THR was not removed from the IPO list until 2020.^30^

### Covariates

We controlled for a continuous indicator for age; a categorical indicator for patient-reported race and ethnicity (non-Hispanic Black, Hispanic, non-Hispanic White, or other; other consisted of Asian, American Indian or Alaska Native, Native Hawaiian or other Pacific Islander, other, or missing); and binary indicators for admission type (inpatient vs outpatient), patient-reported gender (male or female), Medicaid dual-eligibility (a marker for lower socioeconomic status), and 30 Elixhauser comorbid conditions. We also controlled for facility-level categorical indicators for hospital bed size (less than 200, 200 to 400, or more than 400), ownership (government, not-for-profit, for-profit), and DSH payments (in quartiles), and a binary indicator for teaching hospital.

### Statistical Analysis

#### Descriptive Statistics

We report trends in outpatient TKR use from 2016 to 2019. We used χ^2^ and Mann-Whitney *U* tests to compare characteristics of patients undergoing outpatient vs inpatient TKRs.

#### Multivariable Analysis

To determine characteristics of patients who had higher likelihood of undergoing outpatient TKRs, we estimated a multivariable generalized linear mixed model^31^ with logit link that controlled for patient and facility characteristics, and included facility-level random effects to account for clustering of patients within facilities.

We estimated multivariable generalized linear mixed models with logit link (for binary outcomes) or log link (for total cost outcome) and a difference-in-differences strategy at the encounter level to examine the association of IPO policy with the overall change in post-TKR outcomes. Difference-in-differences is an econometric method that is commonly used for policy evaluation and isolates the independent association of the policy with outcomes among the treatment group (TKRs) after controlling for changes in the control group (THRs).^32^ It does this by computing the difference in end points before and after policy implementation in the intervention group and compares this to the difference in the control group. Before estimating the difference-in-differences models, we checked for the parallel trends assumption. This assumption does not require the treatment (TKR) and control (THR) groups to be identical, but that the end points for the treatment group would have evolved in the same way as that in the control group in the absence of the policy. In case of violation of the assumption as evidenced by the covariates for year or procedure-year interaction being statistically significant with *P* < .05, we included an interaction of a continuous specification of the year with the TKR or THR variable, as described previously.^33,34,35^ All models controlled for patient- and facility-level covariates and facility-level random effects, including whether surgery was inpatient or outpatient. We used the -margins- command in Stata to compute adjusted estimates. Statistical analysis was conducted using Stata version 17 (StataCorp). A 2-tailed *P* < .05 was considered statistically significant.

#### Sensitivity Analysis

To test for robustness of our findings, we conducted the following sensitivity analyses. First, we estimated generalized linear mixed models with an identity (instead of logit or log) link to evaluate the association of IPO policy with post-TKR outcomes. Second, we reestimated our original models with facility-level fixed effects rather than random effects. Third, we included encounters for Asian, American Indian or Alaska Native, Native Hawaiian or other Pacific Islander, other, or missing race and ethnicity. Fourth, we reclassified all Medicare TKR encounters after policy implementation with stays less than 2 nights as outpatient due to potential differences in the coding of these variables based on billing codes.

## Results

### Descriptive Analysis

**Figure 1.**
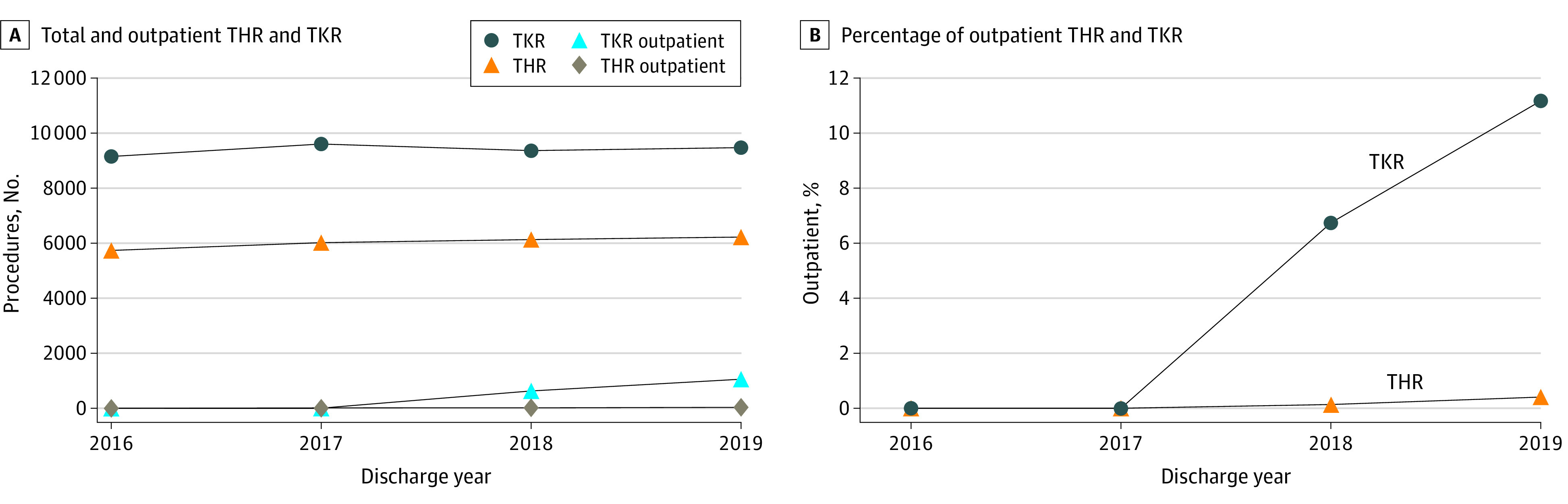
Trends in Total Hip Replacement (THR) and Total Knee Replacement (TKR) from 2016 to 2019

The final cohort included 61 651 index encounters for 55 067 patients (24 085 THRs and 37 566 TKRs) (eFigure in Supplement 1). There were 37 588 TKRs during the study period, with 18 819 performed after removal of TKR from the CMS IPO list. There were increases in the proportion of outpatient TKR over time (629 of 9359 [6.7%] in 2018, 1055 of 9460 [11.2%] in 2019) (Figure 1, Table 1). Patients undergoing outpatient rather than inpatient TKR after IPO policy implementation (2018-2019) had a lower proportion of non-Hispanic Black patients (46 of 1684 [2.7%] vs 936 of 17 135 [5.5%]; *P* < .001), were less commonly female (970 of 1684 [57.6%] vs 11 270 of 17 135 [65.8%]; *P* < .001), and less commonly dually eligible for Medicare and Medicaid (57 of 1684 [3.4%] vs 1251 of 17 135 [7.3%]; *P* < .001) (Table 1). The mean (SD) age of the Medicare TKR cohort in 2018 to 2019 was 73.8 (5.9) years; 12 240 patients [65.0%] were female; 823 [4.4%] were Hispanic, 982 [5.2%] non-Hispanic Black, and 15 714 (83.5%) were non-Hispanic White. A total of 1308 participants (7.0%) were dually eligible for Medicaid. The unadjusted 30-day and 90-day readmission rates for all 18 819 patients undergoing TKR were 0.7% (127 patients) and 1.1% (211 patients); 30-day and 90-day ED visit rates were 1.2% (221 patients) and 1.7% (313 patients), 29.2% (5499 patients) were discharged to a facility, and the mean (SD) cost for the index encounter was $15 398 ($11 925). Hospital characteristics are in eTable 2 in Supplement 1. The comparison of THRs and TKRs before and after IPO policy implementation is in eTable 3 in Supplement 1.

**Table 1.** Characteristics of Medicare Patients Undergoing Total Knee Replacement in the Inpatient and Outpatient Cohorts After the Inpatient-Only Policy Implementation (2018-2019)^a^

Characteristic	Patients, No. (%)			*P *value
	Inpatient (n = 17 135)	Outpatient (n = 1684)	Total (n = 18 819)	
Age, mean (SD), y	73.9 (6.0)	72.9 (5.5)	73.8 (5.9)	<.001
Race and ethnicity				
Hispanic	806 (4.7)	17 (1.0)	823 (4.4)	<.001
Non-Hispanic Black	936 (5.5)	46 (2.7)	982 (5.2)	
Non-Hispanic White	14 159 (82.6)	1555 (92.3)	15 714 (83.5)	
Other^b^	1234 (7.2)	66 (3.9)	1300 (6.9)	
Gender				
Male	5865 (34.2)	714 (42.4)	6579 (35.0)	<.001
Female	11 270 (65.8)	970 (57.6)	12 240 (65.0)	
Dual-eligibility for Medicaid				
Not dually eligible	15 884 (92.7)	1627 (96.6)	17 511 (93.0)	<.001
Dually eligible	1251 (7.3)	57 (3.4)	1308 (7.0)	
Elixhauser comorbidities				
Congestive heart failure	765 (4.5)	36 (2.1)	801 (4.3)	<.001
Cardiac arrhythmias	2722 (15.9)	181 (10.7)	2903 (15.4)	<.001
Valvular disease	1214 (7.1)	90 (5.3)	1304 (6.9)	.01
Pulmonary circulation disorders^c^	NR	NR	NR	<.001
Peripheral vascular disorders	662 (3.9)	36 (2.1)	698 (3.7)	<.001
Hypertension, uncomplicated	10 686 (62.4)	1013 (60.2)	11 699 (62.2)	.07
Paralysis^c^	NR	NR	NR	.48
Other neurological disorders	457 (2.7)	14 (0.8)	471 (2.5)	<.001
Chronic pulmonary disease	2827 (16.5)	193 (11.5)	3020 (16.0)	<.001
Diabetes, uncomplicated	2425 (14.2)	279 (16.6)	2704 (14.4)	.01
Diabetes, complicated	1119 (6.5)	55 (3.3)	1174 (6.2)	<.001
Hypothyroidism	3498 (20.4)	205 (12.2)	3703 (19.7)	<.001
Liver disease^c^	NR	NR	197 (1.0)	.01
Peptic ulcer disease excluding bleeding^c^	NR	NR	60 (0.3)	.02
Lymphoma^c^	NR	NR	73 (0.4)	.83
Metastatic cancer^c^	NR	NR	20 (0.1)	.54
Solid tumor without metastasis^c^	NR	NR	162 (0.9)	.21
Rheumatoid arthritis/collagen vascular diseases	888 (5.2)	44 (2.6)	932 (5.0)	<.001
Coagulopathy	587 (3.4)	16 (1.0)	603 (3.2)	<.001
Obesity	6602 (38.5)	309 (18.3)	6911 (36.7)	<.001
Weight loss^c^	NR	NR	35 (0.2)	.06
Fluid and electrolyte disorders^c^	NR	NR	1710 (9.1)	<.001
Blood loss anemia^c^	NR	NR	260 (1.4)	<.001
Deficiency anemia^c^	NR	NR	267 (1.4)	.001
Alcohol abuse^c^	NR	NR	114 (0.6)	.29
Drug abuse^c^	NR	NR	81 (0.4)	.10
Psychoses^c^	NR	NR	42 (0.2)	.04
Depression	2086 (12.2)	55 (3.3)	2141 (11.4)	<.001
Hypertension, complicated	1815 (10.6)	88 (5.2)	1903 (10.1)	<.001
Patient residential location				
Urban	15 290 (89.2)	1488 (88.4)	16 778 (89.2%)	.41
Rural	1840 (10.7)	196 (11.6)	2036 (10.8%)	
Missing	NR	NR	NR	
Year				
2018	8730 (50.9)	629 (37.4)	9359 (49.7)	<.001
2019	8405 (49.1)	1055 (62.6)	9460 (50.3)	
Unadjusted outcomes				
Readmission <30 d^c^	NR	NR	127 (0.7)	.02
Readmission <90 d^c^	NR	NR	211 (1.1)	.01
ED visit <30 d^c^	NR	NR	221 (1.2)	.01
ED visit <90 d^c^	297 (1.7)	16 (1.0)	313 (1.7)	.02
Non–home discharge	5441 (31.8)	58 (3.4)	5499 (29.2)	<.001
Total cost, mean (SD), $	15 656 (12 295)	12 923 (7022)	15 398 (11 925)	<.001

Abbreviations: ED, emergency department; NR, not reported.

^a^ *P* value from χ^2^ or Mann-Whitney *U* test comparing the distribution of variables across the inpatient and outpatient groups.

^b^ Other race and ethnicity consisted of Asian, American Indian or Alaska Native, Native Hawaiian or other Pacific Islander, other, or missing.

^c^ Masked values in a row with at least 1 cell containing a value of less than 11 per New York Statewide Planning and Research Cooperative System (SPARCS) publication policy.

### Multivariable Analysis

#### Rates of Outpatient vs Inpatient TKR

After IPO implementation, the adjusted rate of outpatient TKR vs inpatient TKR was lower for older patients (age 85 years, 5.99% vs age 65 years, 9.05%; adjusted difference [AD], −3.06%, 95% CI, −4.24% to −1.88%; *P* < .001), non-Hispanic Black patients compared with non-Hispanic White patients (6.30% vs 7.73%; AD, −1.44%; 95% CI, −2.81% to −0.07%; *P* = .048), female compared with male patients (7.29% vs 8.20%; AD −0.91%; 95% CI −1.52% to −0.29%; *P* = .002), and for hospitals receiving higher DSH payments compared with those receiving lower DSH payments (eg, 5.62% vs 21.95% for the second vs first quartile; AD, −16.33%; 95% CI, −28.12% to −4.54%; *P* = .007) (Table 2). Certain medical comorbidities, such as diabetes with complications, also lowered the adjusted rate of outpatient TKR. The adjusted rate of outpatient TKR was higher for large hospitals (with more than 400 beds) compared with small hospitals (less than 200 beds) (11.66% vs 4.02%; AD, 7.64%; 95% CI, 7.64% to 14.80%; *P* = .04) and for patients operated on in 2019 compared with 2018 (9.43% in 2019 vs 5.79% in 2018; AD, 3.64%; 95% CI, 2.66% to 4.62%; *P* < .001).

**Table 2.** Odds of Undergoing TKR in the Outpatient Setting in Medicare Patients After Inpatient-Only Policy Implementation (2018-2019)^a^

Variable	OR (95% CI)	Adjusted percentage of outpatient TKR (95% CI)	Adjusted difference in percentage of outpatient TKR with respect to reference level (95% CI)
Admission type			
Age^b^	0.96 (0.95 to 0.97)^c^	NR	NR
65 y	NR	9.05 (6.04 to 12.05)	[Reference]
75 y	NR	7.40 (4.73 to 10.06)	−1.65 (−2.31 to −0.99)^c^
85 y	NR	5.99 (3.56 to 8.41)	−3.06 (−4.24 to −1.88)^c^
Race and ethnicity			
Non-Hispanic White	1 [Reference]	7.73 (5.00 to 10.46)	[Reference]
Non-Hispanic Black	0.68 (0.46 to 1.00)^d^	6.30 (3.56 to 9.03)	−1.44 (−2.81 to −0.07)^d^
Hispanic	0.79 (0.43 to 1.45)	6.84 (3.50 to 10.18)	−0.89 (−3.12 to 1.34)
Other	0.95 (0.66 to 1.36)	7.51 (4.51 to 10.51)	−0.22 (−1.65 to 1.21)
Gender			
Male	1 [Reference]	8.20 (5.36 to 11.03)	[Reference]
Female	0.80^e^ (0.69 to 0.92)	7.29 (4.64 to 9.94)	−0.91 (−1.52 to −0.29)^e^
Dual-eligibility for Medicaid			
Not dually eligible	1 [Reference]	7.68 (4.97 to 10.40)	[Reference]
Dually eligible	0.79 (0.55 to 1.13)	6.79 (3.98 to 9.59)	−0.90 (−2.21 to 0.42)
Patient residential location			
Urban	1 [Reference]	7.61 (4.90 to 10.31)	[Reference]
Rural	1.05 (0.81 to 1.35)	7.79 (4.93 to 10.64)	0.18 (−0.83 to 1.19)
Elixhauser comorbidities			
Congestive heart failure	0.73 (0.43 to 1.24)	6.50 (3.45 to 9.55)	−1.17 (−3.03 to 0.70)
Cardiac arrhythmias	0.66 (0.53 to 0.82)^c^	6.29 (3.76 to 8.81)	−1.56 (−2.43 to −0.70)^c^
Valvular disease	0.73 (0.53 to 1.00)	6.52 (3.82 to 9.22)	−1.19 (−2.35 to −0.03)^d^
Pulmonary circulation disorders	0.39 (0.13 to 1.19)	4.53 (0.96 to 8.11)	−3.12 (−6.20 to −0.04)^d^
Peripheral vascular disorders	0.62 (0.39 to 0.97)^d^	5.95 (3.19 to 8.72)	−1.73 (−3.27 to −0.19)^d^
Hypertension, uncomplicated	0.75 (0.64 to 0.87)^d^	7.21 (4.58 to 9.84)	−1.17 (−1.84 to −0.49)^e^
Other neurological disorders	0.29 (0.15 to 0.55)^d^	3.86 (1.54 to 6.18)	−3.86 (−5.63 to −2.08)^c^
Chronic pulmonary disease	0.79 (0.64 to 0.98)^f^	6.86 (4.23 to 9.50)	−0.89 (−1.70 to −0.09)^d^
Diabetes, uncomplicated	0.98 (0.81 to 1.19)	7.55 (4.79 to 10.31)	−0.09 (−0.85 to 0.68)
Diabetes, complicated	0.63 (0.43 to 0.92)^f^	6.03 (3.37 to 8.69)	−1.69 (−3.02 to −0.36)^f^
Hypothyroidism	0.60 (0.49 to 0.74)^d^	6.08 (3.62 to 8.54)	−1.90 (−2.73 to −1.06)^d^
Kidney failure	0.63 (0.35 to 1.11)	6.04 (3.07 to 9.01)	−1.70 (−3.66 to 0.26)
Liver disease	0.65 (0.26 to 1.62)	6.06 (2.20 to 9.91)	−1.58 (−4.66 to 1.49)
Lymphoma	1.98 (0.60 to 6.54)	10.72 (3.85 to 17.59)	3.10 (−3.02 to 9.23)
Metastatic cancer	0.44 (0.05 to 4.19)	4.89 (−1.73 to 11.51)	−2.74 (−9.07 to 3.59)
Solid tumor without metastasis	1.23 (0.54 to 2.82)	8.49 (3.96 to 13.02)	0.87 (−2.68 to 4.43)
Rheumatoid arthritis/collagen vascular diseases	0.59^e^ (0.40 to 0.88)	5.84 (3.22 to 8.46)	−1.88 (−3.21 to −0.54)^e^
Coagulopathy	0.47^f^ (0.24 to 0.93)	5.13 (2.23 to 8.02)	−2.56 (−4.61 to −0.51)^f^
Obesity	0.51^d^ (0.42 to 0.60)	5.92 (3.50 to 8.35)	−2.59 (−3.47 to −1.72)^d^
Fluid and electrolyte disorders	0.10^d^ (0.05 to 0.20)	1.99 (0.46 to 3.52)	−6.03 (−8.04 to −4.01)^d^
Blood loss anemia	0.35 (0.03 to 3.76)	4.25 (−2.02 to 10.52)	−3.40 (−9.43 to 2.64)
Deficiency anemia	0.54 (0.24 to 1.18)	5.47 (2.18 to 8.75)	−2.19 (−4.67 to 0.29)
Alcohol abuse	1.14 (0.42 to 3.07)	8.15 (3.18 to 13.13)	0.53 (−3.61 to 4.67)
Drug abuse	0.51 (0.12 to 2.19)	5.28 (0.40 to 10.15)	−2.36 (−6.76 to 2.04)
Depression	0.33 (0.24 to 0.46)^d^	4.36 (2.24 to 6.48)	−3.64 (−4.89 to −2.40)^d^
Hypertension, complicated	0.75 (0.43 to 1.30)	6.60 (3.51 to 9.70)	−1.11 (−3.13 to 0.91)
Hospital size			
<200 beds	1 [Reference]	4.02 (1.51 to 6.53)	[Reference]
200-400 beds	2.42 (0.67 to 8.66)	6.64 (2.92 to 10.37)	2.63 (−1.31 to 6.57)
>400 beds	7.27 (1.36 to 38.94)^f^	11.66 (5.21 to 18.11)	7.64 (0.48 to 14.80)^f^
Teaching hospital			
Nonteaching hospital	1 [Reference]	7.63 (4.70 to 10.57)	[Reference]
Teaching hospital	1.00 (0.23 to 4.39)	7.61 (2.38 to 12.85)	−0.02 (−5.90 to 5.86)
Hospital ownership			
Government	1 [Reference]	9.60 (1.48 to 17.71)	[Reference]
Not-for-profit	0.62 (0.11 to 3.59)	7.53 (4.79 to 10.27)	−2.07 (−10.21 to 6.07)
DSH quartile			
1	1 [Reference]	21.95 (9.02 to 34.89)	[Reference]
2	0.06 (0.01 to 0.29)^d^	5.63 (1.18 to 10.07)	−16.33 (−28.12 to −4.54)^e^
3	0.22 (0.04 to 1.16)	11.29 (5.71 to 16.88)	−10.66 (−23.60 to 2.28)
4	0.03 (0 to 0.30)^e^	3.87 (0.98 to 6.76)	−18.09 (−31.81 to −4.36)^f^
Missing	0.02 (0 to 0.82)^f^	2.71 (−4.00 to 9.43)	−19.24 (−32.54 to −5.94)^e^
Year^c^	2.53 (2.19 to 2.91)^d^		
2018	NR	5.79 (3.42 to 8.16)	[Reference]
2019	NR	9.43 (6.37 to 12.50)	3.64 (2.66 to 4.62)^d^

Abbreviations: DSH, disproportionate share hospital payments; NR, not reported; OR, odds ratio; TKR, total knee replacement.

^a^ Odds of undergoing outpatient surgery after policy implementation was modeled using a generalized linear mixed model with logit link adjusting for patient-level covariates, hospital-level covariates, and hospital random effects. Adjusted rate of outpatient TKR and the difference in the rates were estimated from the same model using the -margins- with contrast command in Stata 17.

^b^ The age and year variables were treated as continuous variables in the analysis. The odds ratios for these variables can be interpreted as an increase/decrease in odds of undergoing outpatient TKRs for each unit increase in the variable (ie, age or year).

^c^ *P* < .001.

^d^ *P* < .05.

^e^ *P* < .01.

#### Association of IPO Policy With Post-TKR Outcomes Relative to Post-THR Outcomes

**Figure 2.**
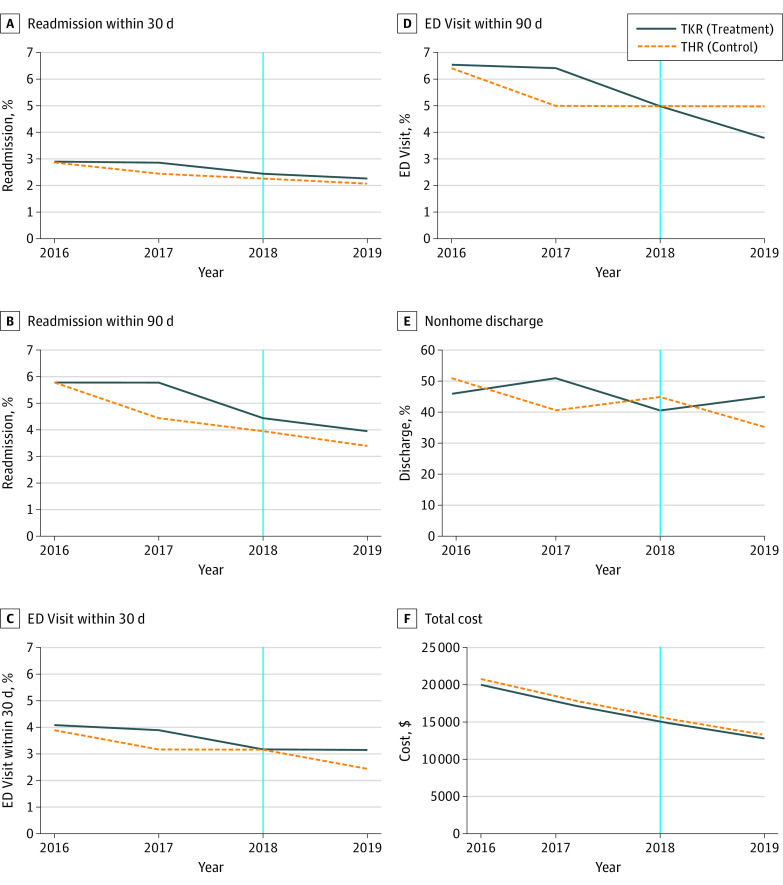
Adjusted Trends in Outcomes Before and After Policy Implementation Data show the analysis of the 2016-2019 New York Statewide Planning and Research Cooperative System Database. The y-axis values represent the adjusted estimates for each outcome during each year. The x-axis values represent the start of the year. The vertical line separates years before and after Medicare’s inpatient-only list policy implementation for total knee replacements on January 1, 2018. ED indicates emergency department; THR, total hip replacement; TKR, total knee replacement.

The parallel trends assumption was violated for 90-day readmissions, 30-day and 90-day ED visits, and non–home discharge end points and outcomes (eTable 4 in Supplement 1). We addressed this violation by including interactions between year and procedure in the difference-in-differences models for these outcomes.^33,34^ The overall TKR rates of postoperative readmissions and ED visits were significantly lower after IPO policy implementation compared with before, while cost was significantly higher (Table 3; Figure 2; full model estimates available in eTable 5 in Supplement 1). Compared with TKRs prior to IPO policy implementation, TKRs after implementation had lower 30-day readmissions (AD, −2.11%; 95% CI, −2.73% to −1.48%; *P* < .001), 90-day readmissions (AD, −3.23%; 95% CI, −4.04% to −2.42%; *P* < .001), 30-day ED visits (AD, −2.45%; 95% CI, −3.17% to −1.72%; *P* < .001), 90-day ED visits (AD, −4.01%; 95% CI, −4.91% to −3.11%; *P* < .001); higher cost (AD, $2988; 95% CI, $415 to $5561; *P* = .03); and no difference in non–home discharge (AD, 0.80%; 95% CI, −1.12% to 2.71%; *P* = .76). However, these changes in the TKR cohort were not significantly different from the changes in the THR cohort other than an increase in cost of $770 per encounter (AD, $770; 95% CI, $83 to $1457; *P* = .03) (Table 3). Our findings from the sensitivity analysis were overall similar to those seen in the main analysis (eTables 6, 7, 8, and 9 in Supplement 1).

**Table 3.** Adjusted Outcomes for THR (Control) and TKR (Treatment) Cohorts Before and After Inpatient-Only Policy Implementation^a^

Outcome	Adjusted estimate, % (95% CI)						
	THR (control) (n = 24 085)^b^			TKR (treatment) (n = 37 566)^c^			IPO effect estimate^d^
	Before estimate	After estimate	Difference	Before estimate	After estimate	Difference	
Readmission <30 d	3.48 (2.82 to 4.15)	1.09 (0.77 to 1.41)	−2.40 (−3.23 to −1.56)^e^	3.13 (2.63 to 3.63)	1.02 (0.76 to 1.30)	−2.11 (−2.73 to −1.48)^e^	0.29 (−0.71 to 1.29)
Readmission <90 d	5.93 (5.09 to 6.77)	2.17 (1.66 to 2.68)	−3.75 (−4.83 to −2.67)^e^	5.19 (4.54 to 5.83)	1.95 (1.54 to 2.37)	−3.23 (−4.04 to −2.42)^e^	0.52 (−0.77 to 0.18)
ED visit <30 d	4.26 (3.63 to 4.89)	1.49 (1.11 to 1.88)	−2.76 (−3.63 to −1.90)^e^	4.11 (3.59 to 4.63)	1.67 (1.33 to 2.00)	−2.45 (−3.17 to −1.72)^e^	0.32 (−0.79 to 1.43)
ED visit <90 d	6.75 (5.93 to 7.56)	2.32 (1.82 to 2.82)	−4.43 (−5.53 to −3.33)^e^	6.54 (5.86 to 7.21)	2.52 (2.09 to 2.96)	−4.01 (−4.91 to −3.11)^e^	0.42 (−0.96 to 1.79)
Non–home discharge	37.11 (33.56 to 40.65)	38.39 (34.76 to 42.02)	1.28 (−1.13 to 3.69)	41.39 (37.84 to 44.95)	42.19 (38.60 to 45.78)	0.80 (−1.12 to 2.71)	−0.49 (−3.56 to 2.59)
Total cost, $ (95% CI)	15 019 (12 477 to 17 560)	17 237 (15 549 to 18 924)	2218 (−36 to 4473)	15 263 (12 701 to 17 824)	18 250 (16 292 to 20 209)	2988 (415 to 5561)^f^	770 (83 to 1457)^f^

Abbreviations: ED, emergency department; IPO, inpatient-only; THR, total hip replacement; TKR, total knee replacement.

^a^ Adjusted outcomes (as percentages for binary outcomes and 2019 US dollars for total cost) from encounter-level models. Binary outcomes were modeled using generalized linear mixed models with a logit link adjusting for patient-level covariates, hospital-level covariates, and hospital random effects. Total cost was modeled using a generalized linear mixed model with clustering by facility, gamma distribution, and log link. The key independent variables were procedure (THR vs TKR), policy phase (before vs after IPO policy implementation), and an interaction between these 2 variables. Full model estimates (odds ratios for binary outcomes and coefficients for total cost models, both with 95% CIs) are included in eTable 5 in Supplement 1. The results of the test for parallel trends are included in eTable 4 in Supplement 1. The outcomes and changes in outcomes were obtained using the Stata margins and contrast commands. Models for 90-day readmissions, 30-day and 90-day ED visits, and non–home discharge outcomes included an interaction of year with procedure (TKR) because of violation of the parallel trends assumption in the preintervention period as shown in eTable 4 in Supplement 1.

^b^ Adjusted outcomes (as percentages for binary outcomes and dollars for total cost) for THRs before implementation (2016-2017), after implementation (2018-2019), and the differences with IPO policy implementation.

^c^ Adjusted outcomes (as percentages for binary outcomes and dollars for total cost) for TKRs before implementation (2016-2017), after implementation (2018-2019), and the differences with IPO policy implementation.

^d^ Difference-in-differences estimates (as percentages for binary outcomes and dollars for total cost) for the association of the policy with outcomes after TKR (treatment) group after controlling for changes in outcomes after THR (control). This is considered the policy effect.

^e^ *P* < .001.

^f^ *P* < .05.

## Discussion

TKR was removed from the CMS IPO list in 2018 due to evidence that these surgeries would be feasible to perform in the outpatient setting for Medicare patients. The association of this policy with TKR utilization and outcomes is largely unknown. We showed that use of outpatient TKR for Medicare patients is on the rise; that older, Black, and female patients, patients with more medically complex conditions, and patients treated in safety-net hospitals were less likely to be selected for outpatient TKR; and that the TKR IPO policy implementation was associated with an overall increase in TKR cost but no difference in postoperative readmissions, ED visits, or non–home discharge.

We found that outpatient TKR utilization has been increasing since policy implementation, and that patient-level and hospital-level factors including patient race and ethnicity are associated with outpatient TKR selection. The increase in outpatient TKR use was expected because TKR was considered an inpatient procedure by CMS prior to IPO policy implementation. Our inferences are consistent with the prior work by Barnes et al^21^ showing that the rate of outpatient TKR in Medicare patients has increased from 0.2% in 2017 to 36.4% in the second quarter of 2019. We found that non-Hispanic Black and female patients were less likely to undergo outpatient TKR after controlling for medical comorbidities and other potential confounding factors. These findings raise questions regarding whether these patients may have reduced access to outpatient TKR and whether there may be a potential disincentive to care for these patients. The reason for this finding is unclear and likely multifactorial. Surgeons may be carrying out inadequate risk assessment in these patients, or these patients may be treated by surgeons who less commonly perform outpatient TKR or are treated at facilities not equipped to support outpatient TKR. This finding raises questions of whether IPO policy implementation may have worsened racial and ethnic and gender disparities in access to TKR.^36,37,38^

Few studies have specifically evaluated the association of IPO implementation with outcomes of TKR in Medicare patients. DeMik et al^39^ reported no change in postoperative readmissions following TKR after IPO implementation compared with the period before implementation in the National Surgical Quality Improvement Program database; however, hospitals self-select into the program and the authors did not focus specifically in Medicare patients, to whom the policy directly applies. Our study fills an important gap by evaluating the policy association with utilization and cost outcomes specifically on Medicare TKRs compared with a control group, and we observed an overall increase in cost for TKRs but no association between IPO implementation and other TKR outcomes in the Medicare population after controlling for secular trends in the THR group. Because outpatient TKRs were shown to have lower overall cost than inpatient TKRs, further increases in the proportion of outpatient TKRs may help to reduce overall increases in per-encounter spending for TKRs.

Our study has important implications for policy, practice, and future research. First, the racial and ethnic disparities associated with outpatient TKR use need to be investigated further and considered in future policies. Next, surgeons should feel comfortable performing TKR in the outpatient setting for appropriately selected patients. Next, the cost implications of IPO policy implementation should be considered in policy making and could be investigated further. Future work may focus on the effect of the 2020 addition of TKR to the CMS ASC Covered Procedures List and removal of THR from IPO list, as well as effect of the recent addition of outpatient TKR to bundled payment programs.^40^

### Strengths and Limitations

Strengths of our study include triangulation of evidence related to IPO policy implementation from multiple angles, including consideration of rates of outpatient TKR, description of patients undergoing outpatient TKR, and the association of the policy with a range of clinical, utilization, and economic outcomes.

Our study had multiple limitations. First, we used administrative claims data from NYS, and our findings were dependent on the presence and reliable coding of variables. We do not have data on potentially important factors including clinical severity of comorbidities or patient preferences, and it is possible that some total joint replacement procedures were performed in facilities not included in the data set. However, the research questions that we investigate can only be addressed using administrative data such as SPARCS that have a broad geographic scope. Moreover, the focus on NYS, which is a demographically diverse state, increases the generalizability of our findings. Second, any readmission or ED visit occurring outside of NYS postoperatively would not be captured. We minimized this by excluding patients who were not NYS residents. Third, we only evaluated cost to the payer at the index encounter, and cost had to be estimated from facility charges. We were unable to track the spending that may have incurred in the postoperative 30-day and 90-day periods and estimate cost from the Medicare fee schedule due to the nature of the SPARCS data.

## Conclusions

In this cohort study of patients undergoing TKR and THR, we found increasing use of outpatient TKR following IPO policy implementation, racial and ethnic disparities in utilization of outpatient TKR, and increased cost but no overall increase in postoperative health care utilization for TKRs relative to THRs with the removal of TKRs from the IPO list. A continual monitoring of the use and outcomes of outpatient TKRs is essential for realizing the policy’s intended goals and avoiding unintended worsening of disparities.

## Notes

Supplement 1. **eFigure.** Case Selection for Study **eTable 1.** Procedure Codes Used to Identify TKR or THR Cases **eTable 2.** Characteristics of Facilities Where TKR Was Performed in Medicare Patients in the Inpatient and Outpatient Cohorts During the Study Period, 2016-2019 **eTable 3.** Characteristics of Medicare Patients in the THR (Control) and TKR (Intervention) Cohorts Before (2016-2017) and After (2018-2019) Inpatient-Only Policy Implementation **eTable 4.** Results From Multivariable Models Examining Parallel Trends in Outcomes for the THR (Control) and TKR (Intervention) Before Inpatient-Only Policy Implementation, 2016-2017 **eTable 5.** Results From Multivariable Models Examining Outcomes During the Period Before (2016-2017) and After (2018-2019) Inpatient-Only Policy Implementation (2018-2019) for Medicare THR and TKR Cases **eTable 6.** Results From Multivariable Generalized Linear Mixed Models With Identity Link (Instead of Logit or Log Link) Examining Outcomes During the Period Before (2016-2017) and After Inpatient-Only Policy Implementation (2018-2019) for Medicare THR and TKR Cases **eTable 7.** Results From Multivariable Models With Fixed Effects for Facility Examining Outcomes During the Period Before (2016-2017) and After Inpatient-Only Policy Implementation (2018-2019) for Medicare THR and TKR Cases **eTable 8.** Results from Multivariable Models Examining Outcomes During the Period Before (2016-2017) After Inpatient-Only Policy Implementation (2018-2019) for Medicare THR and TKR Cases With Alternate Race and Ethnicity Specification **eTable 9.** Results From Multivariable Models Examining Outcomes During the Period Before (2016-2017) and After Inpatient-Only Policy Implementation (2018-2019) for Medicare THR and TKR Cases With Inpatient and Outpatient Status Determined Based on Length of Stay in the Postintervention Period for TKR

Supplement 2.
Data Sharing Statement
