# Supplementary material for: Association of Total Knee Replacement Removal From the Inpatient-Only List With Outpatient Surgery Utilization and Outcomes in Medicare Patients
Source: JAMA Netw Open. 2023 Jun 5;6(6):e2316769. doi: 10.1001/jamanetworkopen.2023.16769 (PMC10242427; doi:10.1001/jamanetworkopen.2023.16769)
Supplement: Supplement 1. — eFigure. Case Selection for Study eTable 1. Procedure Codes Used to Identify TKR or THR Cases eTable 2. Characteristics of Facilities Where TKR Was Performed in Medicare Patients in the Inpatient and Outpatient Cohorts During the Study Period, 2016-2019 eTable 3. Characteristics of Medicare Patients in the THR (Control) and TKR (Intervention) Cohorts Before (2016-2017) and After (2018-2019) Inpatient-Only Policy Implementation eTable 4. Results From Multivariable Models Examining Parallel Trends in Outcomes for the THR (Control) and TKR (Intervention) Before Inpatient-Only Policy Implementation, 2016-2017 eTable 5. Results From Multivariable Models Examining Outcomes During the Period Before (2016-2017) and After (2018-2019) Inpatient-Only Policy Implementation (2018-2019) for Medicare THR and TKR Cases eTable 6. Results From Multivariable Generalized Linear Mixed Models With Identity Link (Instead of Logit or Log Link) Examining Outcomes During the Period Before (2016-2017) and After Inpatient-Only Policy Implementation (2018-2019) for Medicare THR and TKR Cases eTable 7. Results From Multivariable Models With Fixed Effects for Facility Examining Outcomes During the Period Before (2016-2017) and After Inpatient-Only Policy Implementation (2018-2019) for Medicare THR and TKR Cases eTable 8. Results from Multivariable Models Examining Outcomes During the Period Before (2016-2017) After Inpatient-Only Policy Implementation (2018-2019) for Medicare THR and TKR Cases With Alternate Race and Ethnicity Specification eTable 9. Results From Multivariable Models Examining Outcomes During the Period Before (2016-2017) and After Inpatient-Only Policy Implementation (2018-2019) for Medicare THR and TKR Cases With Inpatient and Outpatient Status Determined Based on Length of Stay in the Postintervention Period for TKR [file jamanetwopen-e2316769-s001.pdf]

Supplemental Online Content

Schloemann DT, Sajda T, Ricciardi BF, Thirukumaran CP. Association of total knee replacement removal from the inpatient-only list with outpatient surgery utilization and outcomes in Medicare patients. *JAMA Netw Open.* 2023;6(6):e2316769. doi:10.1001/jamanetworkopen.2023.16769

eFigure 1. Case Selection for Study

eTable 1. Procedure Codes Used to Identify TKR or THR Cases

eTable 2. Characteristics of Facilities Where TKR Was Performed in Medicare Patients in the Inpatient and Outpatient Cohorts During the Study Period, 2016-2019

eTable 3. Characteristics of Medicare Patients in the THR (Control) and TKR (Intervention) Cohorts Before (2016-2017) and After (2018-2019) Inpatient-Only Policy Implementation

eTable 4. Results From Multivariable Models Examining Parallel Trends in Outcomes for the THR (Control) and TKR (Intervention) Before Inpatient-Only Policy Implementation, 2016-2017

eTable 5. Results From Multivariable Models Examining Outcomes During the Period Before (2016-2017) and After (2018-2019) Inpatient-Only Policy Implementation (2018-2019) for Medicare THR and TKR Cases

eTable 6. Results From Multivariable Generalized Linear Mixed Models With Identity Link (Instead of Logit or Log Link) Examining Outcomes During the Period Before (2016-2017) and After Inpatient-Only Policy Implementation (2018-2019) for Medicare THR and TKR Cases

eTable 7. Results From Multivariable Models With Fixed Effects for Facility Examining Outcomes During the Period Before (2016-2017) and After Inpatient-Only Policy Implementation (2018-2019) for Medicare THR and TKR Cases

eTable 8. Results from Multivariable Models Examining Outcomes During the Period Before (2016-2017) After Inpatient-Only Policy Implementation (2018-2019) for Medicare THR and TKR Cases With Alternate Race and Ethnicity Specification

eTable 9. Results From Multivariable Models Examining Outcomes During the Period Before (2016-2017) and After Inpatient-Only Policy Implementation (2018-2019) for

© 2023 Schloemann DT et al. *JAMA Network Open.*

Medicare THR and TKR Cases With Inpatient and Outpatient Status Determined Based on Length of Stay in the Postintervention Period for TKR

This supplemental material has been provided by the authors to give readers additional information about their work.

© 2023 Schloemann DT et al. *JAMA Network Open.*

eFigure 1. Case Selection for Study

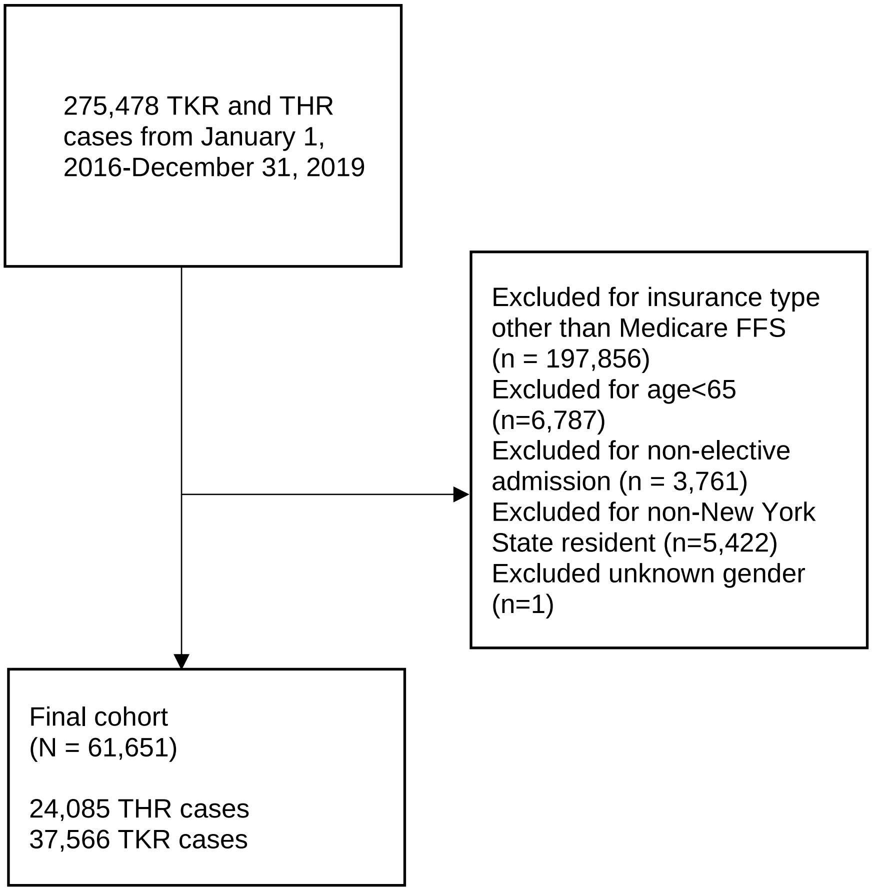

© 2023 Schloemann DT et al. *JAMA Network Open.*

eTable 1. Procedure Codes Used to Identify TKR or THR Cases[a]

Procedure	Code Type	Codes	Description
TKR	CPT	27447	Arthroplasty, knee, condyle and plateau; medial and lateral compartments with or without patella resurfacing (total knee arthroplasty)
TKR	ICD-10-PCS	0SRC069	Replacement of Right Knee Joint with Oxidized Zirconium on Polyethylene Synthetic Substitute, Cemented, Open Approach
TKR	ICD-10-PCS	0SRC06A	Replacement of Right Knee Joint with Oxidized Zirconium on Polyethylene Synthetic Substitute, Uncemented, Open Approach
TKR	ICD-10-PCS	0SRC06Z	Replacement of Right Knee Joint with Oxidized Zirconium on Polyethylene Synthetic Substitute, Open Approach
TKR	ICD-10-PCS	0SRC0J9	Replacement of Right Knee Joint with Synthetic Substitute, Cemented, Open Approach
TKR	ICD-10-PCS	0SRC0JA	Replacement of Right Knee Joint with Synthetic Substitute, Uncemented, Open Approach
TKR	ICD-10-PCS	0SRC0JZ	Replacement of Right Knee Joint with Synthetic Substitute, Open Approach
TKR	ICD-10-PCS	0SRD069	Replacement of Left Knee Joint with Oxidized Zirconium on Polyethylene Synthetic Substitute, Cemented, Open Approach
TKR	ICD-10-PCS	0SRD06A	Replacement of Left Knee Joint with Oxidized Zirconium on Polyethylene Synthetic Substitute, Uncemented, Open Approach
TKR	ICD-10-PCS	0SRD06Z	Replacement of Left Knee Joint with Oxidized Zirconium on Polyethylene Synthetic Substitute, Open Approach
TKR	ICD-10-PCS	0SRD0J9	Replacement of Left Knee Joint with Synthetic Substitute, Cemented, Open Approach
TKR	ICD-10-PCS	0SRD0JA	Replacement of Left Knee Joint with Synthetic Substitute, Uncemented, Open Approach
TKR	ICD-10-PCS	0SRD0JZ	Replacement of Left Knee Joint with Synthetic Substitute, Open Approach
THR	CPT	27130	Arthroplasty, acetabular and proximal femoral prosthetic replacement (total hip arthroplasty), with or without autograft or allograft
THR	ICD-10-PCS	0SR9019	Replacement of Right Hip Joint with Metal Synthetic Substitute, Cemented, Open Approach
THR	ICD-10-PCS	0SR901A	Replacement of Right Hip Joint with Metal Synthetic Substitute, Uncemented, Open Approach
THR	ICD-10-PCS	0SR901Z	Replacement of Right Hip Joint with Metal Synthetic Substitute, Open Approach
THR	ICD-10-PCS	0SR9029	Replacement of Right Hip Joint with Metal on Polyethylene Synthetic Substitute, Cemented, Open Approach
THR	ICD-10-PCS	0SR902A	Replacement of Right Hip Joint with Metal on Polyethylene Synthetic Substitute, Uncemented, Open Approach
THR	ICD-10-PCS	0SR902Z	Replacement of Right Hip Joint with Metal on Polyethylene Synthetic Substitute, Open Approach

Procedure	Code Type	Codes	Description
THR	ICD-10-PCS	0SR9039	Replacement of Right Hip Joint with Ceramic Synthetic Substitute, Cemented, Open Approach
THR	ICD-10-PCS	0SR903A	Replacement of Right Hip Joint with Ceramic Synthetic Substitute, Uncemented, Open Approach
THR	ICD-10-PCS	0SR903Z	Replacement of Right Hip Joint with Ceramic Synthetic Substitute, Open Approach
THR	ICD-10-PCS	0SR9049	Replacement of Right Hip Joint with Ceramic on Polyethylene Synthetic Substitute, Cemented, Open Approach
THR	ICD-10-PCS	0SR904A	Replacement of Right Hip Joint with Ceramic on Polyethylene Synthetic Substitute, Uncemented, Open Approach
THR	ICD-10-PCS	0SR904Z	Replacement of Right Hip Joint with Ceramic on Polyethylene Synthetic Substitute, Open Approach
THR	ICD-10-PCS	0SR9069	Replacement of Right Hip Joint with Oxidized Zirconium on Polyethylene Synthetic Substitute, Cemented, Open Approach
THR	ICD-10-PCS	0SR906A	Replacement of Right Hip Joint with Oxidized Zirconium on Polyethylene Synthetic Substitute, Uncemented, Open Approach
THR	ICD-10-PCS	0SR906Z	Replacement of Right Hip Joint with Oxidized Zirconium on Polyethylene Synthetic Substitute, Open Approach
THR	ICD-10-PCS	0SR90J9	Replacement of Right Hip Joint with Synthetic Substitute, Cemented, Open Approach
THR	ICD-10-PCS	0SR90JA	Replacement of Right Hip Joint with Synthetic Substitute, Uncemented, Open Approach
THR	ICD-10-PCS	0SR90JZ	Replacement of Right Hip Joint with Synthetic Substitute, Open Approach
THR	ICD-10-PCS	0SRB019	Replacement of Left Hip Joint with Metal Synthetic Substitute, Cemented, Open Approach
THR	ICD-10-PCS	0SRB01A	Replacement of Left Hip Joint with Metal Synthetic Substitute, Uncemented, Open Approach
THR	ICD-10-PCS	0SRB01Z	Replacement of Left Hip Joint with Metal Synthetic Substitute, Open Approach
THR	ICD-10-PCS	0SRB029	Replacement of Left Hip Joint with Metal on Polyethylene Synthetic Substitute, Cemented, Open Approach
THR	ICD-10-PCS	0SRB02A	Replacement of Left Hip Joint with Metal on Polyethylene Synthetic Substitute, Uncemented, Open Approach
THR	ICD-10-PCS	0SRB02Z	Replacement of Left Hip Joint with Metal on Polyethylene Synthetic Substitute, Open Approach
THR	ICD-10-PCS	0SRB039	Replacement of Left Hip Joint with Ceramic Synthetic Substitute, Cemented, Open Approach
THR	ICD-10-PCS	0SRB03A	Replacement of Left Hip Joint with Ceramic Synthetic Substitute, Uncemented, Open Approach
THR	ICD-10-PCS	0SRB03Z	Replacement of Left Hip Joint with Ceramic Synthetic Substitute, Open Approach
THR	ICD-10-PCS	0SRB049	Replacement of Left Hip Joint with Ceramic on Polyethylene Synthetic Substitute, Cemented, Open Approach
THR	ICD-10-PCS	0SRB04A	Replacement of Left Hip Joint with Ceramic on Polyethylene Synthetic Substitute, Uncemented, Open Approach
THR	ICD-10-PCS	0SRB04Z	Replacement of Left Hip Joint with Ceramic on Polyethylene Synthetic Substitute, Open Approach

© 2023 Schloemann DT et al. *JAMA Network Open.*

Procedure	Code Type	Codes	Description
THR	ICD-10-PCS	0SRB069	Replacement of Left Hip Joint with Oxidized Zirconium on Polyethylene Synthetic Substitute, Cemented, Open Approach
THR	ICD-10-PCS	0SRB06A	Replacement of Left Hip Joint with Oxidized Zirconium on Polyethylene Synthetic Substitute, Uncemented, Open Approach
THR	ICD-10-PCS	0SRB06Z	Replacement of Left Hip Joint with Oxidized Zirconium on Polyethylene Synthetic Substitute, Open Approach
THR	ICD-10-PCS	0SRB0J9	Replacement of Left Hip Joint with Synthetic Substitute, Cemented, Open Approach
THR	ICD-10-PCS	0SRB0JA	Replacement of Left Hip Joint with Synthetic Substitute, Uncemented, Open Approach
THR	ICD-10-PCS	0SRB0JZ	Replacement of Left Hip Joint with Synthetic Substitute, Open Approach

Abbreviations: THR: Total hip replacement, TKR: Total knee replacement, CPT: Current Procedural Terminology, ICD-10-PCS: International Classification of Diseases, Tenth Modification, Procedure Coding System.

© 2023 Schloemann DT et al. *JAMA Network Open.*

eTable 2. Characteristics of Facilities Where TKR Was Performed in Medicare Patients in the Inpatient and Outpatient Cohorts During the Study Period, 2016-2019[a]

**Characteristic**	**Total**
	N=144
Bed Size: N (%)	
<200 beds	46 (31.9%)
200-400 beds	42 (29.2%)
>400 beds	56 (38.9%)
Teaching Hospital: N (%)	33 (22.9%)
Hospital Ownership: N (%)	
Government	21 (14.6%)
Not-for-profit	123 (85.4%)
DSH Quartile: N (%)	
1	19 (13.2%)
2	38 (26.4%)
3	35 (24.3%)
4	49 (34.0%)

Abbreviations: TKR: Total knee replacement, N: number, %: Row percentage, DSH Quartile: Quartile of disproportionate share hospital payments.

© 2023 Schloemann DT et al. *JAMA Network Open.*

eTable 3. Characteristics of Medicare Patients in the THR and TKR Cohorts Before (2016-2017) and After (2018-2019) Inpatient-Only Policy Implementation[a]

	**Pre-Policy Implementation (2016-2017)**		**Post-Policy Implementation (2018-2019)**	
	**THR**	**TKR**	**THR**	**TKR**
	**N=11,750**	**N=18,747**	**N=12,335**	**N=18,819**
Characteristic				
Admission Type: N (%)				
Inpatient	11,750 (100.0%)	18,745 (100.0%)	12,303 (99.7%)	17,135 (91.1%)
Outpatient			32 (0.3%)	1,684 (8.9%)
Age: Mean (SD)	74.27 (6.57)	73.71 (6.05)	74.10 (6.49)	73.83 (5.92)
Race and Ethnicity: N (%)				
Non-Hispanic Black	475 (4.0%)	991 (5.3%)	431 (3.5%)	982 (5.2%)
Hispanic	538 (4.6%)	1,357 (7.2%)	348 (2.8%)	823 (4.4%)
Non-Hispanic White	10,111 (86.1%)	14,850 (79.2%)	11,035 (89.5%)	15,714 (83.5%)
Other	626 (5.3%)	1,549 (8.3%)	521 (4.2%)	1,300 (6.9%)
Gender: N (%)				
Male	4,410 (37.5%)	6,413 (34.2%)	4,710 (38.2%)	6,579 (35.0%)
Female	7,340 (62.5%)	12,334 (65.8%)	7,625 (61.8%)	12,240 (65.0%)
Dual-Eligibility Status: N (%)				
Not Dually-Eligible	11,221 (95.5%)	17,208 (91.8%)	11,753 (95.3%)	17,511 (93.0%)
Dually-Eligible	529 (4.5%)	1,539 (8.2%)	582 (4.7%)	1,308 (7.0%)
Elixhauser Comorbidities: N (%)				
Congestive Heart Failure	460 (3.9%)	700 (3.7%)	513 (4.2%)	801 (4.3%)
Cardiac Arrhythmias	1,860 (15.8%)	2,849 (15.2%)	1,917 (15.5%)	2,903 (15.4%)
Valvular Disease	906 (7.7%)	1,299 (6.9%)	963 (7.8%)	1,304 (6.9%)
Pulmonary Circulation Disorders	168 (1.4%)	228 (1.2%)	146 (1.2%)	235 (1.2%)
Peripheral Vascular Disorders	535 (4.6%)	734 (3.9%)	557 (4.5%)	698 (3.7%)
Hypertension, uncomplicated	7,138 (60.7%)	12,264 (65.4%)	7,147 (57.9%)	11,699 (62.2%)
Paralysis	§	11 (0.1%)	§	§
Other Neurological Disorders	276 (2.3%)	498 (2.7%)	269 (2.2%)	471 (2.5%)
Chronic Pulmonary Disease	1,747 (14.9%)	2,911 (15.5%)	1,883 (15.3%)	3,020 (16.0%)
Diabetes, Uncomplicated	1,432 (12.2%)	3,078 (16.4%)	1,315 (10.7%)	2,704 (14.4%)
Diabetes, Complicated	387 (3.3%)	866 (4.6%)	573 (4.6%)	1,174 (6.2%)

© 2023 Schloemann DT et al. *JAMA Network Open.*

	Pre-Policy Implementation (2016-2017)		Post-Policy Implementation (2018-2019)	
	THR	TKR	THR	TKR
	N=11,750	N=18,747	N=12,335	N=18,819
Hypothyroidism	2,108 (%)	3,696 (%)	2,314 (%)	3,703 (%)
Liver Disease	111 (0.9%)	221 (1.2%)	126 (1.0%)	197 (1.0%)
Peptic Ulcer Disease Excluding Bleeding	39 (0.3%)	81 (0.4%)	55 (0.4%)	60 (0.3%)
Lymphoma	75 (0.6%)	50 (0.3%)	54 (0.4%)	73 (0.4%)
Metastatic Cancer	40 (0.3%)	24 (0.1%)	41 (0.3%)	20 (0.1%)
Solid Tumor Without Metastasis	108 (0.9%)	119 (0.6%)	138 (1.1%)	162 (0.9%)
Rheumatoid Arthritis/Collagen Vascular Diseases	519 (4.4%)	927 (4.9%)	571 (4.6%)	932 (5.0%)
Coagulopathy	436 (3.7%)	644 (3.4%)	425 (3.4%)	603 (3.2%)
Obesity	3,209 (27.3%)	6,546 (34.9%)	3,539 (28.7%)	6,911 (36.7%)
Weight Loss	30 (0.3%)	14 (0.1%)	69 (0.6%)	35 (0.2%)
Fluid and Electrolyte Disorders	1,230 (10.5%)	1,895 (10.1%)	1,117 (9.1%)	1,710 (9.1%)
Blood Loss Anemia	106 (0.9%)	171 (0.9%)	120 (1.0%)	260 (1.4%)
Deficiency Anemia	130 (1.1%)	250 (1.3%)	161 (1.3%)	267 (1.4%)
Alcohol Abuse	95 (0.8%)	117 (0.6%)	104 (0.8%)	114 (0.6%)
Drug Abuse	31 (0.3%)	53 (0.3%)	61 (0.5%)	81 (0.4%)
Psychoses	26 (0.2%)	39 (0.2%)	20 (0.2%)	42 (0.2%)
Depression	1,314 (11.2%)	2,165 (11.5%)	1,394 (11.3%)	2,141 (11.4%)
Hypertension, Complicated	846 (7.2%)	1,459 (7.8%)	1,187 (9.6%)	1,903 (10.1%)
Patient Residential Location: N (%)				
Urban	10,661 (90.7%)	16,863 (90.0%)	10,982 (89.0%)	16,778 (89.2%)
Rural	1,087 (9.3%)	1,882 (10.0%)	1,351 (11.0%)	2,036 (10.8%)
Unadjusted Outcomes: N (%)				
Readmission with 30 Days	425 (3.6%)	650 (3.5%)	99 (0.8%)	127 (0.7%)
Readmission with 90 Days	780 (6.6%)	1,139 (6.1%)	173 (1.4%)	211 (1.1%)
ED Visit within 30 Days	591 (5.0%)	879 (4.7%)	128 (1.0%)	221 (1.2%)
ED Visit within 90 Days	924 (7.9%)	1,421 (7.6%)	188 (1.5%)	313 (1.7%)
Non-Home Discharge	4,078 (34.7%)	7,901 (42.1%)	3,086 (25.0%)	5,499 (29.2%)
Total Cost ($)	17311 (10618)	17686.61 (10941)	14467 (11555)	15398 (11925)

Abbreviations: THR: Total hip replacement, TKR: Total knee replacement, IPO: Inpatient-only, N: Number, SD: Standard deviation, %: Row percentage, $: 2019 United States Dollars, P: P-value, ED:

© 2023 Schloemann DT et al. *JAMA Network Open.*

Emergency Department, SPARCS: New York Statewide Planning and Research Cooperative System, § indicates masked values in a row with at least one cell containing a value of less than 11 per SPARCS publication policy.

© 2023 Schloemann DT et al. *JAMA Network Open.*

eTable 4. Results From Multivariable Models Examining Parallel Trends in Outcomes for the THR (Control) and TKR (Intervention) Before Inpatient-Only Policy Implementation, 2016-2017[a]

Characteristic	**Readmission within 30 Days** N=30,492	**Readmission within 90 Days** N=30,492	**ED Visit within 30 Days** N=30,496	**ED Visit within 90 Days** N=30,496	**Non-Home Discharge** N=30,496	**Total Cost** N=28,520
	OR (95% CI)	OR (95% CI)	OR (95% CI)	OR (95% CI)	OR (95% CI)	Exponentiated Coefficient (95% CI)
Procedure						
THR	Reference	Reference	Reference	Reference	Reference	Reference
TKR	1.00 (0.67 - 1.49)	0.96 (0.71 - 1.29)	0.75 (0.54 - 1.05)	0.82 (0.63 - 1.08)	1.53*** (1.29 - 1.81)	-0.05 (-0.13 - 0.04)
Year[b]	0.86 (0.71 - 1.05)	0.77*** (0.66 - 0.89)	0.76** (0.64 - 0.90)	0.74*** (0.64 - 0.85)	0.71*** (0.65 - 0.78)	-0.33 (-0.79 - 0.12)
Procedure#Year[b] Interaction						
THR#Year[b]	Reference	Reference	Reference	Reference	Reference	Reference
TKR#Year[b]	0.97 (0.75 - 1.24)	0.96 (0.79 - 1.17)	1.15 (0.93 - 1.43)	1.1 (0.92 - 1.31)	0.9 (0.81 - 1.01)	0.04 (-0.03 - 0.12)

*** indicates P<0.001, ** indicates P<0.01, * indicates P<0.05.

Abbreviations: THR: Total hip replacement, TKR: Total knee replacement, OR: Odds ratio, CI: Confidence interval, P: P-value, ED: Emergency Department.

a: The encounter-level models evaluated outcomes for the period before Inpatient-only (IPO) policy implementation in Medicare patients (2016-2017). Binary outcomes were modeled using generalized linear mixed models with logit link, and adjusting for patient-level covariates, hospital-

© 2023 Schloemann DT et al. *JAMA Network Open.*

level covariates, and hospital random effects. Total cost was modeled using a generalized linear mixed model with clustering by facility, gamma distribution, and log link.

Only the odds ratios and confidence intervals for the procedure, discharge year, and procedure-discharge year interactions are shown. These models also controlled for patient- and facility-level covariates, and facility random effects.

© 2023 Schloemann DT et al. *JAMA Network Open.*

eTable 5. Results From Multivariable Models Examining Outcomes During the Period Before (2016-2017) and After (2018-2019) Inpatient-Only Policy Implementation (2018-2019) for Medicare THR and TKR Cases[a]

**Characteristic**	**Readmission within 30 Days** N=61,637	**Readmission within 90 Days** N=61,637	**ED Visit within 30 Days** N=61,648	**ED Visit within 90 Days** N=61,648	**Non-Home Discharge** N=61,648	**Total Cost** N=57,634
	OR (95% CI)	OR (95% CI)	OR (95% CI)	OR (95% CI)	OR (95% CI)	Exponentiated Coefficient (95% CI)
Procedure						
THR	Reference	Reference	Reference	Reference	Reference	Reference
TKR	0.94 (0.83 to 1.07)	1 (0.76 - 1.31)	0.87 (0.64 - 1.18)	0.93 (0.73 - 1.19)	1.36*** (1.20 - 1.55)	0.02 (-0.04 to 0.07)
Policy Phase						
Pre-IPO List Removal	Reference	Reference	Reference	Reference	Reference	Reference
Post-IPO List Removal	0.33*** (0.24 to 0.44)	0.35*** (0.25 - 0.47)	0.34*** (0.24 - 0.48)	0.32*** (0.24 - 0.43)	1.08 (0.94 - 1.23)	0.14 (-0.01 to 0.29)
Policy Phase#Procedure Interaction						
Post-IPO List Removal#TKR	0.92 (0.69 to 1.24)	1.03 (0.68 - 1.56)	1.16 (0.73 - 1.83)	1.13 (0.78 - 1.64)	0.97 (0.82 - 1.16)	0.04* (0.01 to 0.08)
Year[b]	0.81*** (0.72 to 0.90)	0.75*** (0.66 - 0.86)	0.76*** (0.66 - 0.89)	0.75*** (0.66 - 0.84)	0.74*** (0.70 - 0.79)	-0.15 (-0.31 to 0.02)
Yearb#Procedure Interaction						
Year[b]#TKR		0.94 (0.79 - 1.12)	1.05 (0.86 - 1.27)	1.02 (0.87 - 1.19)	0.97 (0.90 - 1.05)	
Admission Type						
Inpatient	Reference	Reference	Reference	Reference	Reference	Reference
Outpatient	0.38 (0.14 to 1.02)	0.48* (0.23 - 0.98)	0.43* (0.22 - 0.85)	0.60* (0.36 - 1.00)	0.16*** (0.12 - 0.20)	-0.18 (-0.40 to 0.04)
Age[b]	1.05*** (1.04 to 1.06)	1.04*** (1.03 - 1.05)	1.02*** (1.02 - 1.03)	1.03*** (1.02 - 1.03)	1.11*** (1.10 - 1.11)	0 (-0.00 to 0.00)

© 2023 Schloemann DT et al. *JAMA Network Open.*

Characteristic	**Readmission within 30 Days** N=61,637	**Readmission within 90 Days** N=61,637	**ED Visit within 30 Days** N=61,648	**ED Visit within 90 Days** N=61,648	**Non-Home Discharge** N=61,648	**Total Cost** N=57,634
	OR (95% CI)	OR (95% CI)	OR (95% CI)	OR (95% CI)	OR (95% CI)	Exponentiated Coefficient (95% CI)
Race and Ethnicity						
Non-Hispanic White	Reference	Reference	Reference	Reference	Reference	Reference
Non-Hispanic Black	1.21 (0.93 to 1.57)	1.21 (0.99 to 1.47)	1.09 (0.86 to 1.36)	1.25* (1.04 to 1.49)	1.45*** (1.32 to 1.59)	0.05 (-0.00 to 0.10)
Hispanic	0.72* (0.52 to 0.99)	0.74* (0.58 to 0.95)	0.87 (0.68 to 1.12)	0.98 (0.80 to 1.20)	1.05 (0.95 to 1.17)	-0.17* (-0.33 to -0.02)
Other	1.11 (0.88 to 1.40)	0.99 (0.82 to 1.20)	0.98 (0.80 to 1.19)	0.95 (0.81 to 1.13)	1.04 (0.96 to 1.14)	-0.13 (-0.27 to 0.01)
Gender						
Male	Reference	Reference	Reference	Reference	Reference	Reference
Female	0.83** (0.74 to 0.94)	0.84*** (0.76 - 0.92)	0.82*** (0.74 - 0.90)	0.85*** (0.78 - 0.93)	1.74*** (1.67 - 1.81)	0 (-0.01 to 0.01)
Dual-Eligibility for Medicaid						
Not Dually-Eligible	Reference	Reference	Reference	Reference	Reference	Reference
Dually-Eligible	0.95 (0.75 to 1.20)	1.07 (0.90 - 1.27)	1.24* (1.03 - 1.50)	1.35*** (1.16 - 1.56)	2.11*** (1.95 - 2.29)	0.07* (0.01 to 0.12)
Patient Residential Location						
Urban	Reference	Reference	Reference	Reference	Reference	Reference
Rural	0.81 (0.65 to 1.01)	0.86 (0.73 - 1.03)	0.98 (0.82 - 1.16)	1.06 (0.92 - 1.22)	0.87** (0.80 - 0.95)	-0.11 (-0.23 to 0.02)
Missing			9.82** (1.95 - 49.32)	6.49* (1.27 - 33.09)	0.55 (0.10 - 2.93)	-0.04 (-0.29 to 0.21)
Elixhauser Comorbidities						
Congestive Heart Failure	1.28 (1.00 to 1.64)	1.35** (1.12 - 1.64)	1.26 (1.00 - 1.60)	1.39*** (1.15 - 1.68)	1.61*** (1.44 - 1.79)	0.14*** (0.10 to 0.17)
Cardiac Arrhythmias	1.40*** (1.22 to 1.61)	1.41*** (1.26 - 1.57)	1.05 (0.92 - 1.20)	1.05 (0.95 - 1.17)	1.29*** (1.22 - 1.36)	0.06*** (0.04 to 0.08)
Valvular Disease	1.1 (0.90 to 1.33)	1.12 (0.97 - 1.30)	1.03 (0.86 - 1.23)	1.1 (0.95 - 1.26)	1.03 (0.95 - 1.10)	-0.01 (-0.04 to 0.01)

© 2023 Schloemann DT et al. *JAMA Network Open.*

Characteristic	Readmission within 30 Days N=61,637	Readmission within 90 Days N=61,637	ED Visit within 30 Days N=61,648	ED Visit within 90 Days N=61,648	Non-Home Discharge N=61,648	Total Cost N=57,634
	OR (95% CI)	OR (95% CI)	OR (95% CI)	OR (95% CI)	OR (95% CI)	Exponentiated Coefficient (95% CI)
Pulmonary Circulation Disorders	1.16 (0.79 to 1.71)	1.37* (1.03 - 1.81)	1.3 (0.91 - 1.86)	1.29 (0.97 - 1.72)	1.19* (1.01 - 1.41)	0.16*** (0.09 to 0.22)
Peripheral Vascular Disorders	1.06 (0.83 to 1.35)	1.16 (0.97 - 1.39)	0.98 (0.78 - 1.23)	1.01 (0.84 - 1.21)	1.22*** (1.11 - 1.35)	0 (-0.04 to 0.05)
Hypertension, uncomplicated	1.15* (1.00 to 1.33)	1.12* (1.01 - 1.24)	1.02 (0.91 - 1.14)	0.99 (0.91 - 1.09)	1.03 (0.99 - 1.08)	0.02 (-0.01 to 0.05)
Paralysis	4.10* (1.14 to 14.70)	4.68** (1.62 - 13.55)	1.16 (0.15 - 8.80)	3.13* (1.01 - 9.68)	2.46 (0.93 - 6.53)	0.24 (-0.04 to 0.51)
Other Neurological Disorders	1.51** (1.13 to 2.02)	1.27* (1.00 - 1.61)	1.27 (0.97 - 1.66)	1.34** (1.08 - 1.66)	2.72*** (2.42 - 3.06)	0.10*** (0.06 to 0.15)
Chronic Pulmonary Disease	1.26** (1.09 to 1.45)	1.24*** (1.11 - 1.38)	1.06 (0.93 - 1.21)	1.12* (1.01 - 1.24)	1.29*** (1.22 - 1.36)	0.03* (0.01 to 0.05)
Diabetes, Uncomplicated	1.12 (0.96 to 1.30)	1.04 (0.92 - 1.17)	1.06 (0.92 - 1.21)	1.13* (1.02 - 1.26)	1.30*** (1.23 - 1.38)	0.04** (0.01 to 0.06)
Diabetes, Complicated	1.33* (1.05 to 1.68)	1.31** (1.09 - 1.58)	1.07 (0.85 - 1.35)	1.05 (0.87 - 1.27)	1.63*** (1.49 - 1.78)	0.09** (0.03 to 0.14)
Hypothyroidism	0.91 (0.78 to 1.05)	0.91 (0.81 - 1.02)	1.01 (0.89 - 1.14)	1.02 (0.92 - 1.13)	1.05* (1.00 - 1.11)	0.01 (-0.01 to 0.03)
Renal Failure	1.22 (0.87 to 1.71)	1.22 (0.93 - 1.58)	1.22 (0.88 - 1.68)	1.43** (1.10 - 1.85)	1.26*** (1.10 - 1.43)	0.02 (-0.05 to 0.08)
Liver Disease	2.19*** (1.48 to 3.22)	1.94*** (1.42 - 2.65)	1.03 (0.65 - 1.63)	1.06 (0.74 - 1.53)	1.31** (1.10 - 1.57)	0.12*** (0.06 to 0.18)
Peptic Ulcer Disease Excluding Bleeding	1.08 (0.47 to 2.46)	1.24 (0.68 - 2.25)	1.31 (0.67 - 2.59)	1.67* (1.02 - 2.75)	1.38* (1.03 - 1.85)	-0.04 (-0.12 to 0.04)
Lymphoma	0.87 (0.38 to 1.98)	0.98 (0.54 - 1.79)	1.48 (0.82 - 2.69)	1.15 (0.67 - 1.96)	1.02 (0.77 - 1.36)	0.09** (0.02 to 0.16)
Metastatic Cancer	0.87 (0.26 to 2.92)	1.73 (0.84 - 3.55)	1.69 (0.74 - 3.86)	1.4 (0.69 - 2.83)	1.57* (1.03 - 2.40)	0.09 (-0.01 to 0.18)
Solid Tumor Without Metastasis	1.17 (0.65 to 2.11)	1.04 (0.66 - 1.65)	1.02 (0.61 - 1.72)	1.17 (0.78 - 1.75)	1 (0.80 - 1.23)	-0.02 (-0.07 to 0.03)

© 2023 Schloemann DT et al. *JAMA Network Open.*

Characteristic	Readmission within 30 Days N=61,637	Readmission within 90 Days N=61,637	ED Visit within 30 Days N=61,648	ED Visit within 90 Days N=61,648	Non-Home Discharge N=61,648	Total Cost N=57,634
	OR (95% CI)	OR (95% CI)	OR (95% CI)	OR (95% CI)	OR (95% CI)	Exponentiated Coefficient (95% CI)
Rheumatoid Arthritis/Collagen Vascular Diseases	1.16 (0.91 to 1.49)	1.19 (0.99 - 1.44)	1.17 (0.95 - 1.44)	1.17 (0.99 - 1.39)	1.31*** (1.20 - 1.43)	0.02 (-0.01 to 0.05)
Coagulopathy	1.15 (0.87 to 1.53)	1.26* (1.03 - 1.56)	1.23 (0.96 - 1.57)	1.17 (0.96 - 1.44)	1.19** (1.07 - 1.31)	-0.01 (-0.12 to 0.10)
Obesity	1.18* (1.03 to 1.35)	1.13* (1.02 - 1.25)	0.91 (0.81 - 1.02)	0.93 (0.85 - 1.02)	1.54*** (1.47 - 1.61)	-0.12*** (-0.19 to -0.05)
Weight Loss	1.19 (0.42 to 3.35)	1.17 (0.53 - 2.60)	0.25 (0.03 - 1.83)	1.16 (0.52 - 2.55)	1.83** (1.26 - 2.64)	0.44*** (0.18 to 0.69)
Fluid and Electrolyte Disorders	1.24* (1.04 to 1.48)	1.28*** (1.12 - 1.46)	1.24** (1.06 - 1.44)	1.27*** (1.12 - 1.44)	1.36*** (1.28 - 1.45)	0.11** (0.04 to 0.17)
Blood Loss Anemia	0.85 (0.48 to 1.51)	0.97 (0.63 - 1.48)	1.29 (0.84 - 2.00)	1.19 (0.82 - 1.72)	1.02 (0.85 - 1.22)	-0.01 (-0.11 to 0.10)
Deficiency Anemia	1.11 (0.72 to 1.72)	1.19 (0.86 - 1.66)	1.02 (0.68 - 1.53)	1.12 (0.82 - 1.54)	1.13 (0.97 - 1.33)	0.04 (-0.02 to 0.09)
Alcohol Abuse	0.64 (0.31 to 1.31)	1.02 (0.65 - 1.59)	0.9 (0.51 - 1.58)	0.84 (0.53 - 1.34)	1.34** (1.08 - 1.68)	0.01 (-0.06 to 0.08)
Drug Abuse	2.57** (1.32 to 4.98)	1.6 (0.87 - 2.93)	1.17 (0.54 - 2.51)	1.38 (0.77 - 2.46)	1.43* (1.06 - 1.95)	0.12* (0.00 to 0.23)
Psychoses	1.53 (0.61 to 3.89)	1.9 (0.98 - 3.68)	1.27 (0.55 - 2.95)	1.23 (0.63 - 2.42)	4.58*** (2.91 - 7.19)	0.05 (-0.08 to 0.18)
Depression	1.31** (1.11 to 1.55)	1.42*** (1.25 - 1.61)	1.15 (0.99 - 1.34)	1.30*** (1.16 - 1.46)	1.41*** (1.33 - 1.49)	0 (-0.03 to 0.02)
Hypertension, Complicated	1.3 (0.92 to 1.83)	1.16 (0.89 - 1.52)	0.82 (0.59 - 1.14)	0.71** (0.54 - 0.92)	1 (0.88 - 1.14)	0.06 (-0.01 to 0.13)
Bed Size						
<200 beds	Reference	Reference	Reference	Reference	Reference	Reference
200-400 beds	0.89 (0.69 to 1.14)	0.93 (0.75 - 1.16)	0.95 (0.78 - 1.14)	0.96 (0.81 - 1.15)	0.97 (0.63 - 1.47)	-0.43** (-0.71 to -0.16)
>400 beds	1.01 (0.74 to 1.37)	0.88 (0.67 - 1.16)	1.01 (0.80 - 1.27)	0.91 (0.73 - 1.13)	1.06 (0.62 - 1.78)	-0.42** (-0.70 to -0.13)

© 2023 Schloemann DT et al. *JAMA Network Open.*

Characteristic	Readmission within 30 Days N=61,637	Readmission within 90 Days N=61,637	ED Visit within 30 Days N=61,648	ED Visit within 90 Days N=61,648	Non-Home Discharge N=61,648	Total Cost N=57,634
	OR (95% CI)	OR (95% CI)	OR (95% CI)	OR (95% CI)	OR (95% CI)	Exponentiated Coefficient (95% CI)
Teaching Hospital						
Non-Teaching Hospital	Reference	Reference	Reference	Reference	Reference	Reference
Teaching Hospital	1.05 (0.80 to 1.38)	1.16 (0.92 - 1.48)	0.82* (0.67 - 1.00)	0.93 (0.77 - 1.12)	0.89 (0.56 - 1.44)	-0.21 (-0.52 to 0.10)
Hospital Ownership						
Government	Reference	Reference	Reference	Reference	Reference	Reference
Not-for-profit	1.11 (0.74 to 1.67)	0.98 (0.70 - 1.38)	0.81 (0.60 - 1.09)	0.85 (0.65 - 1.11)	1.79* (1.06 - 3.01)	0.21 (-0.07 to 0.49)
DSH Quartile						
1	Reference	Reference	Reference	Reference	Reference	Reference
2	1.11 (0.78 to 1.58)	1.27 (0.93 - 1.72)	1.21 (0.92 - 1.60)	1.01 (0.79 - 1.29)	0.53* (0.31 - 0.91)	0.23 (-0.11 to 0.56)
3	0.96 (0.66 to 1.40)	1.08 (0.78 - 1.49)	1.08 (0.81 - 1.45)	0.95 (0.73 - 1.24)	0.46** (0.26 - 0.83)	0.2 (-0.13 to 0.52)
4	0.9 (0.58 to 1.40)	1.06 (0.72 - 1.56)	1.06 (0.76 - 1.49)	0.98 (0.72 - 1.34)	0.75 (0.39 - 1.43)	0.62* (0.14 to 1.09)
Missing	1.27 (0.66 to 2.43)	1.13 (0.63 - 2.05)	1.11 (0.67 - 1.83)	0.95 (0.59 - 1.51)	0.41 (0.11 - 1.49)	-0.49** (-0.79 to -0.19)

*** indicates P<0.001, ** indicates P<0.01, * indicates P<0.05.

Abbreviations: TKR: Total knee replacement, DSH Quartile: Quartile of disproportionate share hospital payments, OR: Odds ratio, CI: Confidence interval, P: P-value, ED: Emergency department, N: Number of encounters in specified group.

a: The encounter-level models evaluated outcomes for the period before (2016-2017) and after Inpatient-only (IPO) policy implementation in Medicare patients (2018-2019). Binary outcomes were modeled using generalized linear mixed models with logit link, and adjusting for patient-level covariates, hospital-level covariates, and hospital random effects. Total cost was modeled using a generalized linear mixed model with clustering by facility, gamma distribution, and log link. Models for 90-day readmissions, 30- and 90-day ED visits, and non-home discharge

© 2023 Schloemann DT et al. *JAMA Network Open.*

outcomes included an interaction of year with procedure (TKR) because of violation of the parallel trends assumption in the pre-intervention period as shown in eTable 4.

b: The age and year variables were treated as continuous variables in the analysis. The odds ratios for these variables can be interpreted as an increase/decrease in odds of undergoing outpatient TKRs for each unit increase in the variable (i.e., age or year).

© 2023 Schloemann DT et al. *JAMA Network Open.*

eTable 6. Results From Multivariable Generalized Linear Mixed Models With Identity Link (Instead of Logit or Log Link) Examining Outcomes During the Period Before (2016-2017) and After Inpatient-Only Policy Implementation (2018-2019) for Medicare THR and TKR Cases[a]

Characteristic	Readmission within 30 Days N=61,651	Readmission within 90 Days N=61,651	ED Visit within 30 Days N=61,651	ED Visit within 90 Days N=61,651	Non-Home Discharge N=61,651	Total Cost ($) N=57,634
	Coefficient (95% CI)	Coefficient (95% CI)	Coefficient (95% CI)	Coefficient (95% CI)	Coefficient (95% CI)	Coefficient (95% CI)
Procedure						
THR	Reference	Reference	Reference	Reference	Reference	Reference
TKR	0 (-0.01 to 0.00)	0.00 (-0.01 to 0.01)	-0.01 (-0.01 to 0.00)	0.00 (-0.02 to 0.01)	0.07*** (0.05 to 0.09)	58.07 (-137.09 to 253.23)
Policy Phase						
Pre-IPO List Removal	Reference	Reference	Reference	Reference	Reference	Reference
Post-IPO List Removal	-0.02*** (-0.02 to -0.01)	-0.03*** (-0.04 to -0.02)	-0.02*** (-0.03 to -0.01)	-0.04*** (-0.05 to -0.03)	0.01 (-0.01 to 0.04)	2502.19*** (2163.50 to 2840.87)
Policy Phase#Procedure Interaction						
Post-IPO List Removal#TKR	0 (-0.00 to 0.00)	0.01 (-0.01 to 0.02)	0.00 (-0.01 to 0.01)	0.00 (-0.01 to 0.02)	0 (-0.03 to 0.03)	377.68** (103.63 to 651.73)

Abbreviations: TKR: Total knee replacement, DSH Quartile: Quartile of disproportionate share hospital payments, CI: Confidence interval, P: P-value, ED: Emergency department, N: Number of encounters in specified group.

a: The encounter-level models evaluated outcomes for the period before (2016-2017) and after Inpatient-only (IPO) policy implementation in Medicare patients (2018-2019). All outcomes were modeled using multivariable generalized linear mixed models with identify link, and adjusting

© 2023 Schloemann DT et al. *JAMA Network Open.*

for patient-level covariates, hospital-level covariates, and hospital random effects. Models for 90-day readmissions, 30- and 90-day ED visits, and non-home discharge outcomes included an interaction of year with procedure (TKR) because of violation of the parallel trends assumption in the pre-intervention period. *** indicates P<0.001, ** indicates P<0.01, * indicates P<0.05.

© 2023 Schloemann DT et al. *JAMA Network Open.*

eTable 7. Results From Multivariable Models With Fixed Effects for Facility Examining Outcomes During the Period Before (2016-2017) and After Inpatient-Only Policy Implementation (2018-2019) for Medicare THR and TKR Cases[a].

Characteristic	**Readmission within 30 Days** N=61,637	**Readmission within 90 Days** N=61,637	**ED Visit within 30 Days** N=61,648	**ED Visit within 90 Days** N=61,648	**Non-Home Discharge** N=61,648	**Total Cost** N=57,634
	OR (95% CI)	OR (95% CI)	OR (95% CI)	OR (95% CI)	OR (95% CI)	Exponentiated Coefficient (95% CI)
Procedure						
THR	Reference	Reference	Reference	Reference	Reference	Reference
TKR	0.95 (0.83 to 1.08)	1.01 (0.77 to 1.32)	0.93 (0.72 to 1.19)	0.93 (0.72 to 1.19)	1.36*** (1.20 to 1.55)	0.00 (-0.01 to 0.01)
Policy Phase						
Pre-IPO List Removal	Reference	Reference	Reference	Reference	Reference	Reference
Post-IPO List Removal	0.33*** (0.24 to 0.45)	0.35*** (0.26 to 0.48)	0.33*** (0.24 to 0.44)	0.33*** (0.24 to 0.44)	1.08 (0.94 to 1.24)	0.07*** (0.05 to 0.09)
Policy Phase#Procedure Interaction						
Post-IPO List Removal#TKR	0.92 (0.68 to 1.23)	1.03 (0.68 to 1.56)	1.11 (0.76 to 1.61)	1.11 (0.76 to 1.61)	0.97 (0.81 to 1.15)	0.03*** (0.01 to 0.04)

Abbreviations: TKR: Total knee replacement, DSH Quartile: Quartile of disproportionate share hospital payments, OR: Odds ratio, CI: Confidence interval, P: P-value, ED: Emergency department, N: Number of encounters in specified group.

a: The encounter-level models evaluated outcomes for the period before (2016-2017) and after Inpatient-only (IPO) policy implementation in Medicare patients (2018-2019). Binary outcomes were modeled using generalized linear mixed models with logit link, and adjusting for patient-level covariates, hospital-level covariates, and hospital fixed effects. Total cost was modeled using a generalized linear mixed model with facility fixed effects, gamma distribution, and log link. Models for 90-day readmissions, 30- and 90-day ED visits, and non-home discharge outcomes

© 2023 Schloemann DT et al. *JAMA Network Open.*

included an interaction of year with procedure (TKR) because of violation of the parallel trends assumption in the pre-intervention period as shown in eTable 4. *** indicates P<0.001, ** indicates P<0.01, * indicates P<0.05.

© 2023 Schloemann DT et al. *JAMA Network Open.*

eTable 8. Results from Multivariable Models Examining Outcomes During the Period Before (2016-2017) After Inpatient-Only Policy Implementation (2018-2019) for Medicare THR and TKR Cases With Alternate Race and Ethnicity Specification[a]

**Characteristic**	**Readmission within 30 Days** N=61,637	**Readmission within 90 Days** N=61,637	**ED Visit within 30 Days** N=61,648	**ED Visit within 90 Days** N=61,648	**Non-Home Discharge** N=61,648	**Total Cost** N=57,634
	OR (95% CI)	OR (95% CI)	OR (95% CI)	OR (95% CI)	OR (95% CI)	Exponentiated Coefficient (95% CI)
Procedure						
THR	Reference	Reference	Reference	Reference	Reference	Reference
TKR	0.95 (0.83 to 1.08)	1 (0.76 to 1.31)	0.93 (0.73 to 1.19)	0.93 (0.73 to 1.19)	1.36*** (1.20 to 1.55)	0.02 (-0.04 to 0.07)
Policy Phase						
Pre-IPO List Removal	Reference	Reference	Reference	Reference	Reference	Reference
Post-IPO List Removal	0.33*** (0.24 to 0.45)	0.35*** (0.25 to 0.47)	0.32*** (0.24 to 0.43)	0.32*** (0.24 to 0.43)	1.08 (0.94 to 1.24)	0.14 (-0.01 to 0.29)
Policy Phase#Procedure Interaction						
Post-IPO List Removal#TKR	0.92 (0.68 to 1.23)	1.03 (0.68 to 1.55)	1.13 (0.78 to 1.65)	1.13 (0.78 to 1.65)	0.97 (0.82 to 1.16)	0.04* (0.01 to 0.08)

Abbreviations: TKR: Total knee replacement, DSH Quartile: Quartile of disproportionate share hospital payments, OR: Odds ratio, CI: Confidence interval, P: P-value, ED: Emergency department, N: Number of encounters in specified group.

a: The encounter-level models evaluated outcomes for the period before (2016-2017) and after Inpatient-only (IPO) policy implementation in Medicare patients (2018-2019). Binary outcomes were modeled using generalized linear mixed models with logit link, and adjusting for patient-level covariates, hospital-level covariates, and hospital random effects. Total cost was modeled using a generalized linear mixed model with clustering by facility, gamma distribution, and log link. Models for 90-day readmissions, 30- and 90-day ED visits, and non-home discharge

© 2023 Schloemann DT et al. *JAMA Network Open.*

outcomes included an interaction of year with procedure (TKR) because of violation of the parallel trends assumption in the pre-intervention period as shown in eTable 4. *** indicates P<0.001, ** indicates P<0.01, * indicates P<0.05. Race and ethnicity was specified as a categorical variable (non-Hispanic White, non-Hispanic Black, Hispanic, Asian, American Indian or Alaska Native, Native Hawaiian or Other Pacific Islander, Other, or Missing).

© 2023 Schloemann DT et al. *JAMA Network Open.*

eTable 9. Results From Multivariable Models Examining Outcomes During the Period Before (2016-2017) and After Inpatient-Only Policy Implementation (2018-2019) for Medicare THR and TKR Cases With Inpatient and Outpatient Status Determined Based on Length of Stay in the Postintervention Period for TKR[a]

**Characteristic**	**Readmission within 30 Days** N=61,637	**Readmission within 90 Days** N=61,637	**ED Visit within 30 Days** N=61,648	**ED Visit within 90 Days** N=61,648	**Non-Home Discharge** N=61,648	**Total Cost** N=57,634
	OR (95% CI)	OR (95% CI)	OR (95% CI)	OR (95% CI)	OR (95% CI)	Exponentiated Coefficient (95% CI)
Procedure						
THR	Reference	Reference	Reference	Reference	Reference	Reference
TKR	0.94 (0.83 to 1.07)	0.99 (0.76 to 1.30)	0.87 (0.64 to 1.18)	0.93 (0.73 to 1.19)	1.31*** (1.15 to 1.49)	0.02 (-0.04 to 0.07)
Policy Phase						
Pre-IPO List Removal	Reference	Reference	Reference	Reference	Reference	Reference
Post-IPO List Removal	0.32*** (0.24 to 0.44)	0.35*** (0.25 to 0.47)	0.34*** (0.24 to 0.48)	0.32*** (0.24 to 0.43)	1.07 (0.93 to 1.23)	0.13 (-0.02 to 0.29)
Policy Phase#Procedure Interaction						
Post-IPO List Removal#TKR	1.00 (0.74 to 1.35)	1.07 (0.71 to 1.62)	1.16 (0.73 to 1.83)	1.11 (0.76 to 1.61)	1.08 (0.91 to 1.29)	0.06** (0.02 to 0.10)

Abbreviations: TKR: Total knee replacement, DSH Quartile: Quartile of disproportionate share hospital payments, OR: Odds ratio, CI: Confidence interval, P: P-value, ED: Emergency department, N: Number of encounters in specified group.

© 2023 Schloemann DT et al. *JAMA Network Open.*

a: The encounter-level models evaluated outcomes for the period before (2016-2017) and after Inpatient-only (IPO) policy implementation in Medicare patients (2018-2019). Binary outcomes were modeled using generalized linear mixed models with logit link, and adjusting for patient-level covariates, hospital-level covariates, and hospital random effects. Total cost was modeled using a generalized linear mixed model with clustering by facility, gamma distribution, and log link. Models for 90-day readmissions, 30- and 90-day ED visits, and non-home discharge outcomes included an interaction of year with procedure (TKR) because of violation of the parallel trends assumption in the pre-intervention period as shown in eTable 4. *** indicates P<0.001, ** indicates P<0.01, * indicates P<0.05. All TKR stays were coded as outpatient stays if less than two nights during the period after policy implementation.

© 2023 Schloemann DT et al. *JAMA Network Open.*
