# Supplementary material for: Association of Total Knee Replacement Removal From the Inpatient-Only List With Outpatient Surgery Utilization and Outcomes in Medicare Patients
Source: JAMA Netw Open. 2023 Jun 5;6(6):e2316769. doi: 10.1001/jamanetworkopen.2023.16769 (PMC10242427; doi:10.1001/jamanetworkopen.2023.16769)
Supplement: Supplement 2. — Data Sharing Statement [file jamanetwopen-e2316769-s002.pdf]

Data Sharing Statement

Schloemann. Association of Total Knee Replacement Removal from the Inpatient-Only List with Outpatient Surgery Utilization and Outcomes in Medicare Patients. *JAMA Netw Open*. Published June 05, 2023. doi:10.1001/jamanetworkopen.2023.16769

Data
Data available: No

Additional Information
Explanation for why data not available: Not permitted by data source due to patient privacy constraints.
